# Improvement in coronary microvascular dysfunction evaluated by cardiac magnetic resonance in patients with hypertrophic obstructive cardiomyopathy after transapical beating-heart septal myectomy

**DOI:** 10.3389/fcvm.2023.1233004

**Published:** 2023-10-24

**Authors:** Yun Zhao, Lu Huang, Chenhe Li, Dazhong Tang, Yi Luo, Chunlin Xiang, Xiaoyue Zhou, Jing Fang, Xiang Wei, Liming Xia

**Affiliations:** ^1^ Department of Radiology, Tongji Hospital, Tongji Medical College, Huazhong University of Science and Technology, Wuhan, China; ^2^ Department of Cardiovascular Surgery, Tongji Hospital, Tongji Medical College, Huazhong University of Science and Technology, Wuhan, China; ^3^ MR Collaboration, Siemens Healthineers Ltd., Shanghai, China

**Keywords:** Coronary microvascular dysfunction, myocardial perfusion, obstructive hypertrophic cardiomyopathy, cardiac magnetic resonance, transapical beating-heart septal myectomy

## Abstract

**Background:**

Coronary microvascular dysfunction (CMD) is a pathophysiological mechanism underlying hypertrophic obstructive cardiomyopathy (HOCM). However, few studies have investigated the potential effect of transapical beating-heart septal myectomy (TA-BSM) on coronary microvascular function. This study aimed to evaluate coronary microvascular function in HOCM after TA-BSM using cardiac magnetic resonance (CMR) and to investigate the determinants of improvement in coronary microvascular dysfunction.

**Materials and methods:**

28 patients with HOCM who underwent TA-BSM were prospectively enrolled in this study from March 2022 to April 2023. All patients received CMR before and after TA-BSM. CMR-derived parameters were compared, including the maximum wall thickness, native T1 value, T2 value, late gadolinium enhancement (LGE), and perfusion indexes (Slope_max_, Time_max_, and Sl_max_). Univariate and multivariate linear regression identified variables associated with the rate of Slope_max_ change.

**Results:**

Compared with the preoperative parameters, left ventricular function and myocardial perfusion were significantly improved after TA-BSM (all *P* < 0.05), although still lower than in healthy controls. In the analysis of the myocardial perfusion parameter rate of change, the rate of Slope_max_ change was the most significant (*P* = 0.002) in HOCM. In the multivariable regression analysis, age (adjusted *β* = 0.551), weight of the resected myocardium (adjusted *β* = 0.191), maximum wall thickness (adjusted *β* = −0.406), LGE (adjusted *β* = 0.260), and Δ left ventricular outflow tract (LVOT) pressure gradient (adjusted *β* = −0.123) were significantly associated with the rate of Slope_max_ change in HOCM (*P* < 0.05 for all).

**Conclusion:**

Coronary microvascular dysfunction in both hypertrophic and non-hypertrophic myocardial segments was improved in patients after TA-BSM. Microcirculatory perfusion evaluated by CMR can be a potential tool to evaluate the improvement of CMD in HOCM.

## Introduction

Hypertrophic cardiomyopathy (HCM) is the most common inherited cardiovascular disease presenting with segmental myocardial hypertrophy (1). Obstructive hypertrophic cardiomyopathy (HOCM) is a subtype of HCM characterized by septal hypertrophy and left ventricular outflow tract (LVOT) obstruction. Coronary microvascular dysfunction (CMD) is one of the most critical pathophysiologic changes in HOCM, associated with various clinical features such as malignant ventricular tachyarrhythmia and sudden cardiac death (SCD) (2). Previous studies have shown that LVOT obstruction and CMD independently predict sudden death (3, 4). Myocardial hyper-dynamicity, a disorganized myocardial arrangement, and interstitial fibrosis can cause structural arteriole changes, leading to an inadequate blood supply. Chronic and recurrent ischemia can result in progressive fibrosis, eventually leading to left ventricular remodeling and heart failure (HF) (5).

According to the latest guidelines (6), surgical septal myectomy is the most effective treatment for symptoms and obstruction relief in HOCM. Transapical beating-heart septal myectomy (TA-BSM) (7) is a new, minimally invasive septal surgery for HOCM treatment. TA-BSM uses a set of atherectomy devices to perform septal myectomy without extracorporeal circulation in a state where the heart is beating. As a new surgical procedure, myocardial microcirculatory perfusion can improve the multidimensional efficacy evaluation system and comprehensively assess the treatment effect.

Cardiac magnetic resonance (CMR), a unique tissue characterization technology, has become a powerful tool for evaluating HCM patients. CMR can accurately assess myocardial morphology and microvascular function due to its high temporal and spatial resolution (8). In addition, CMR first-pass perfusion and late gadolinium enhancement (LGE) imaging can be used to assess myocardial perfusion and fibrosis quantitatively (9). Although the presence and prognostic impact of microvascular impairment in HCM patients has been confirmed in previous studies, it is unclear whether the myocardial microcirculatory environment improves after myectomy. Therefore, this study aimed to evaluate improvement in the coronary microcirculation in HOCM patients using CMR.

## Materials and methods

### Study design and participants

Twenty-eight patients with HOCM who underwent TA-BSM at Tongji Hospital from March 2022 to April 2023 were prospectively enrolled in this study. All patients met the clinical diagnosis of HCM according to published guidelines (6): ventricular wall thickness ≥15 mm in one or more segments or maximum wall thickness ≥13 mm in relatives in the absence of another disease that could account for the hypertrophy. HOCM was defined as a left ventricular outflow tract gradient (LVOTG) ≥ 30 mmHg at rest or provoked LVOTG ≥ 50 mmHg. The indications for TA-BSM were mainly (1) severe symptoms, with syncope or near-syncope despite the effectiveness of medical therapy; (2) rest or provoked LVOTG ≥ 50 mmHg; (3) age ≥ 12 years. The major exclusions were: (1) failure to undergo complete MRI examinations (e.g., a previous pacemaker or metal stent implantation, postoperative adverse events, and missed appointments); (2) interval of preoperative CMR before TA-BSM greater than 6 months and interval of postoperative CMR after TA-BSM less than 3 months; (3) concomitant myocardial infarction or coronary artery stenosis ≥ 50% at coronary angiography or computed tomographic angiography (CTA); (4) abnormal subendocardial perfusion ≥ 1 segment corresponding to a coronary vascular distribution; (5) comorbid with other diseases (congenital heart disease, valvular disease, and hypertension); (6) previous cardiac surgery, including alcohols septal ablation, percutaneous radiofrequency ablation, and surgical myectomy; (7) uninterpretable images for perfusion analysis. In addition, 21 healthy individuals matched for age and sex were included as the control group.

### Cardiovascular magnetic resonance data acquisition

All patients underwent a standard cardiac MRI on a 3 T MRI system (MAGNETOM Skyra, Siemens Healthcare, Erlangen, Germany). The CMR protocol included short-axis cine, native T1 mapping, T2 mapping, first-pass perfusion, and late gadolinium-enhanced (LGE) imaging. Cine images were acquired with ECG gating and breath-holding using a segmented, balanced, steady-state free-precession sequence. The typical parameters were: section thickness = 8 mm, section gap = 2 mm, echo time = 1.39 ms, repetition time = 3.2 ms, field of view = 360 × 360 mm^2^, matrix size = 189 × 154, and flip angle = 46°. T1 mapping images were acquired using the modified look-locker recovery sequence with 5b(3b)3b (b for heartbeat) scheme at the left ventricular basal, mid, and apical levels. The acquisition parameters were: echo time = 1.2 ms; repetition time = 2.8 ms, inversion time = 197 ms, increase step = 80 ms, flip angle = 35°, field of view = 360 × 324 mm^2^, matrix = 257 × 232, and slice thickness = 5 mm. The parameters for T2 mapping are as follows: echo time = 1.35 ms; flip angle = 12°, field of view = 360 × 323 mm^2^, matrix = 189 × 170 mm, and slice thickness = 5 mm, T2 preparation duration = 0, 30, 55 ms. The CMR perfusion image used a gradient echo sequence with 0.3 mmol/kg of gadolinium-based contrast agent (Adobenate Dimeglumine, Berlin, Germany). In addition, the scan parameters were: matrix size = 211 × 181, field of view = 380 × 326 mm^2^, slice thickness = 8 mm, repetition time = 2.0 ms, echo time = 0.97 ms, flip angle = 18°, and nominal inversion time = 130 ms. LGE used a phase-sensitive, inverse recovery sequence 10–15 min after contrast injection. The scan parameters were: matrix size = 192 × 173, field of view = 350 × 263 mm^2^, slice thickness = 6.0 mm, repetition time = 3.2 ms, echo time = 1.27 ms, flip angle = 10°, and nominal inversion time = 255 ms.

### Cardiovascular magnetic resonance images analysis

CMR analysis was performed using CVI 42 software (version 5.14.0, Circle Cardiovascular Imaging Inc., Canada). The left atrial (LA) anteroposterior and LA left–right diameters were obtained on three- and four- chamber cine images, respectively. The maximum LA volume (LAV_max_) and minimum LA volume (LAV_min_) were acquired with a combination of two- and four-chamber cine images at the end of ventricular systole and diastole. The LA ejection fraction (LAEF) was calculated as follows: LAEF = [(LAV_max_−LAV_min_) / LAV_max_] × 100%. Left ventricular (LV) structure and functional parameters were measured on short-axis cines, including the maximum wall thickness, LV ejection fraction (LVEF), LV end-diastolic volume (LVEDV), LV end-systolic volume (LVESV), stroke volume (SV), cardiac output (CO) and LV mass (LVM). Some parameters were normalized to body surface area: LAV_max_ index, LAV_min_ index, LV end-diastolic volume index (LVEDVi), cardiac index (CI) and LV mass index (LVMi). In addition, the percent weight of the resected myocardium was calculated by dividing the surgically removed myocardial mass by the mass of the left ventricular myocardium at end-diastole.

**Figure 1:**
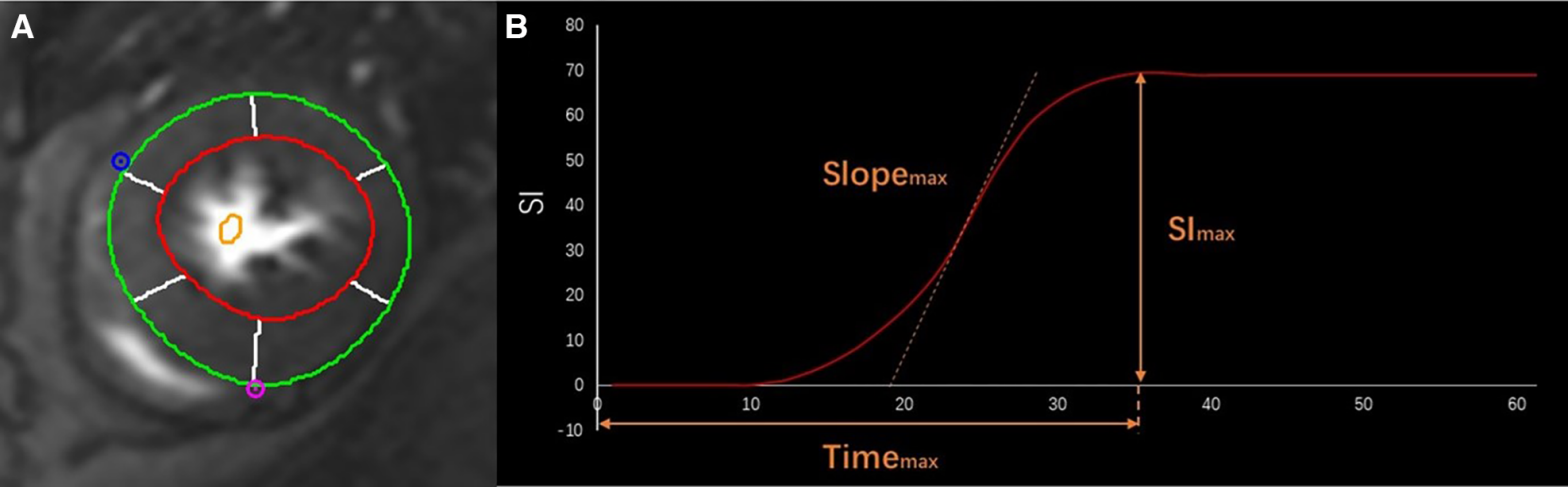
CMR first-pass perfusion contours. The endo- (red curve) and epi-cardial (green curve) borders on the cine images of the short axis (**A**) Curve of time-intensity derived Slope_max_, Time_max_, and SI_max_ in different phases in a healthy person (**B**).

The myocardium was divided into sixteen American Heart Association (AHA) segments for the perfusion assessment. Time-intensity curves were acquired for each segment through the entire perfusion process. The time-to-maximal signal intensity (time_max_), maximal upload of myocardial intensity enhancement (Slope_max_), and maximal signal intensity (Sl_max_) were measured (Figure 1). Strengthening myocardium was identified when LGE was analyzed as the range of a signal threshold ≥ five standard deviations of reference myocardium. Lastly, the native myocardial T1 and T2 values were measured using three slices generating T1/T2 maps from the base to the apex.

### Statistical analysis

Statistical data analysis was conducted using SPSS 25.0 software (IBM SPSS Inc., Chicago, USA). The measurement data conforming to the normal distribution were expressed as means ± standard deviation (*x* ± s). The data at baseline and follow-up were compared using the paired t-test. Data that did not conform to the normal distribution were indicated by medians and quartile M (Q1, Q3), and the data before and after surgery were compared using the paired Wilcoxon signed-rank test. The Pearson correlation coefficient or Spearman's rank correlation coefficient (r) was used to assess the association of continuous variables.

Changes in each variable were represented by △, equaling the preoperative value minus the postoperative value. Potential variables associated with Slope_max_ and Sl_max_ were analyzed by univariate analysis. The variables with *p* < 0.05 by univariate analysis were inputted into the multivariate model as covariates. We randomly selected 20 subjects to assess the intra- and inter-observer reproducibility of left ventricular function, perfusion, and histological parameters by Bland–Altman analysis. Lastly, a *p*-value < 0.05 was considered statistically significant.

## Results

### Patient characteristics

Twenty-eight HOCM patients were enrolled, including fourteen males and seven females, aged 12∼72 years. Twenty-four (85.7%) patients had asymmetric septal hypertrophy, and four (14.3%) had apical hypertrophy. The maximal LV wall thickness was 22.8 ± 1.1 mm. In total, 448 segments were analyzed. Perfusion, native T1 mapping and T2 mapping images were interpretable in all segments. Notably, the mean weight of the HOCM resected myocardium was 6.7 ± 0.6 g. Clinically, the primary symptom experienced by the patients was dyspnea (71.4%). The baseline characteristics of HOCM patients are shown in Table 1.

**Table 1:** Demographic characteristics and clinical parameters associated with TA-BSM.

Parameters	HOCM (*n* = 28)
Age (y)	48.4 ± 3.0
Male [*n* (%)]	20 (71.4)
Body surface area (m^2^)	1.9 ± 0.05
T2DM [*n* (%)]	1 (3.6)
Chronic renal failure [*n* (%)]	1 (3.6)
Family history of HCM [*n* (%)]	6 (21.4)
Myocardial bridge [*n* (%)]	5 (17.9)
Weight of resected myocardium (*g*)	6.7 ± 0.6
6-minute walking distance (m)	316.9 ± 21.4
Score of the Kansas City Cardiomyopathy Questionnaire	63.0 ± 2.8
Symptom [*n* (%)]	
Chest pain	14 (50.0)
Dyspnea	20 (71.4)
Syncope	4 (14.3)
Amaurosis	6 (21.4)
Palpitation	13 (46.4)
Angina	3 (10.7)
Postprandial aggravation of symptom	21 (75.0)
NYHA class	
Ⅰ	0 (0)
Ⅱ	10 (35.7)
Ⅲ	16 (57.1)
Ⅳ	2 (7.2)
Medication use [*n* (%)]	
*β*-blockers	18 (64.3)
Diltiazem	13 (46.4)
Mavacamten / Aficamten	
Biochemistry	
NT-proBNP (pg/ml)	1,119 (481, 1,772)
cTnI (ng/ml)	32.3 (13.5, 141.9)
CK-MB (ng/ml)	1.5 (1.3, 2.5)
Electrocardiogram	
ST-T abnormality [*n* (%)]	24 (85.7)
Rv5 + Sv1 (mV)	4.7 ± 0.4

T2DM, Type 2 diabetes mellitus; HCM, hypertrophic cardiomyopathy; NYHA, New York Heart Association.

**Figure 2:**
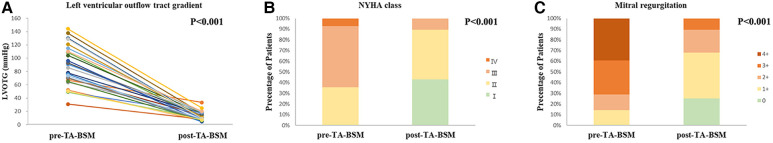
Preoperative and postoperative changes in the left ventricular outflow tract pressure gradient (**A**), NYHA classes (**B**), and mitral regurgitation degree (**C**).

Clinical, ECG, and laboratory data were collected one week before and three months after TA-BSM. All participants experienced relief of symptoms after TA-BSM, and LVOTG was significantly reduced (87.2 ± 5.8 mmHg vs. 13.2 ± 1.3 mmHg, *p* < 0.001) (Figure 2A). At follow-up, we found that the LVOTG increased in some patients three months after surgery relative to the immediate postoperative pressure gradient, within the normal range. In addition, the preoperative cardiac function of eighteen patients (64.3%) was classified as NYHA grade Ⅲ or Ⅳ, while the proportion after surgery was reduced to 10.7% (Figure 2B). Finally, the number of participants with grade 2 and below mitral regurgitation increased from eight (28.6%) to twenty-five (89.2%) (Figure 2C).

## Cardiovascular magnetic resonance analysis

### Left atrial structure and function

Comparisons of the preoperative and postoperative LA parameters are shown in Table. 2. Postoperative LA anteroposterior diameter, LA left-right diameter, LAV_max_ index, and LAV_min_ index were decreased, while LAEF was increased.

**Table 2:** LA parameters in control group and HOCM group before and after TA-BSM.

Parameters	HOCM		*P*-value	Controls	*P*-value for Pre-TA-BSM vs. controls
	Preoperative	Postoperative			
LA anteroposterior diameter (mm)	43.2 ± 1.3	37.7 ± 1.2	<0.001	2.9 ± 0.7	<0.001
LA left-right diameter (mm)	50.2 ± 1.3	42.4 ± 1.1	<0.001	3.6 ± 1.0	<0.001
LAV_max_ index (ml/m^2^)	52.6 (30.4, 78.6)	37.5 ± 3.4	<0.001	31.4 ± 3.5	<0.001
LAV_min_ index (ml/m^2^)	39.0 ± 2.9	27.5 ± 2.5	<0.001	24.3 ± 2.4	<0.001
LAEF (%)	48.9 ± 1.5	53.8 ± 2.0	<0.001	59.4 ± 2.1	<0.001

LAEF, left atrial ejection fraction; LAV, left atrial volume.

### Left ventricular structure and function

The pre- and postoperative parameters of left ventricular function in the control and study groups are listed in Table 3. The end-diastolic wall thickness of the left ventricle was significantly reduced after TA-BSM (22.8 mm vs. 17.7 mm, *p* < 0.001). In addition, LVEF, LVMi, SV, CI, native T1 value, T2 value and LGE volume were decreased (all *p* < 0.05), while LVMi, native T1 value, and T2 value were higher than the control group (all *p* < 0.001).

**Table 3:** LV parameters in control group and HOCM group before and after TA-BSM.

Parameters	HOCM		*P*-value	Controls	*P*-value for Pre-TA-BSM vs. controls
	Preoperative	Postoperative			
LVEF (%)	66.4 ± 1.3	58.5 ± 1.5	<0.001	64.1 ± 1.2	<0.001
LVMi (g/m^2^)	102.7 ± 5.5	85.5 ± 5.2	<0.001	39.5 ± 1.2	<0.001
LVEDVi (ml/m^2^)	83.7 ± 2.7	83.7 ± 2.3	0.712	69.5 ± 2.9	<0.001
LVESVi (ml/m^2^)	28.3 ± 1.6	34.5 ± 1.6	<0.001	25.2 ± 1.6	<0.001
SV (ml)	105.4 ± 4.7	94.2 ± 4.8	0.001	47.6 ± 4.2	<0.001
CI (l/min/m^2^)	3.7 ± 0.2	3.0 ± 0.1	<0.001	2.9 ± 0.2	0.001
Maximal wall thickness (mm)	22.8 ± 1.1	17.7 ± 1.1	<0.001	7.6 ± 1.2	<0.001
Native T1 value (ms)	1,295 ± 2.4	1,278 ± 2.6	<0.001	1,226 ± 2.6	<0.001
T2 value (ms)	40.0 ± 0.1	38.2 ± 0.1	<0.001	34.1 ± 0.1	0.001
Slope_max_ (SI/s)	3.1 (2.4, 4.4)	2.9 (2.3, 3.9)	<0.001	4.7 ± 0.1	<0.001
Time_max_ (s)	43.4 (38.0, 47.1)	36.5 (31.5, 40.1)	<0.001	34.2 ± 0.3	<0.001
Sl_max_	45.0 (33.6, 59.2)	52.2 (40.4, 67.6)	<0.001	55.0 ± 1.3	<0.001
LGE Volume (%)	20.1 ± 2.3	13.6 (5.8, 23.7)	<0.001	-	-

LVEF, left ventricular ejection fraction; LVMi, left ventricular mass index; LVEDVi, left ventricular end-diastolic volume index; LVESVi, left ventricular end-systolic volume index; SV, stroke volume; CI, cardiac Index; Slope_max_, maximal upslope of myocardial intensity enhancement; Time_max_, time to maximal signal intensity; Sl_max_, maximal signal intensity; LGE, late gadolinium enhancement.

### Left ventricular myocardial perfusion

**Figure 3:**
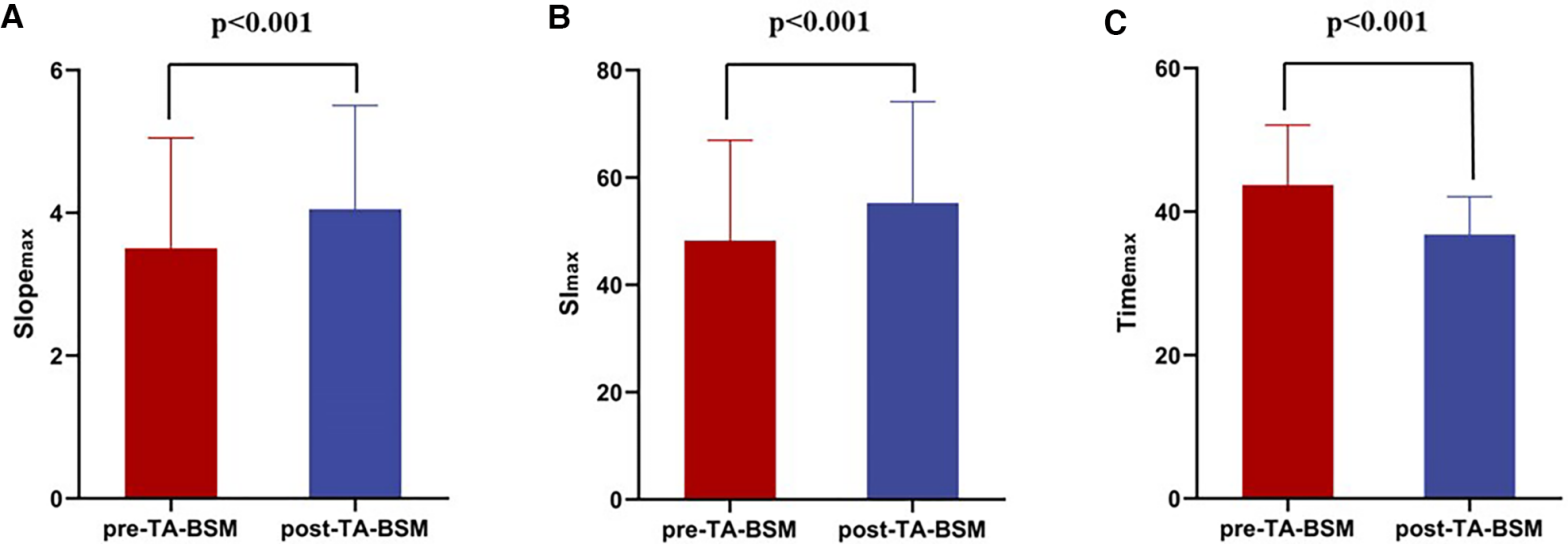
Preoperative and postoperative comparisons of slope_max_ (**A**), sI_max_ (**B**), and time_max_ (**C**) of the myocardial segments. The Slope_max_ and SI_max_ were statistically significantly elevated after TA-BSM and Time_max_ were lowered.

**Figure 4:**
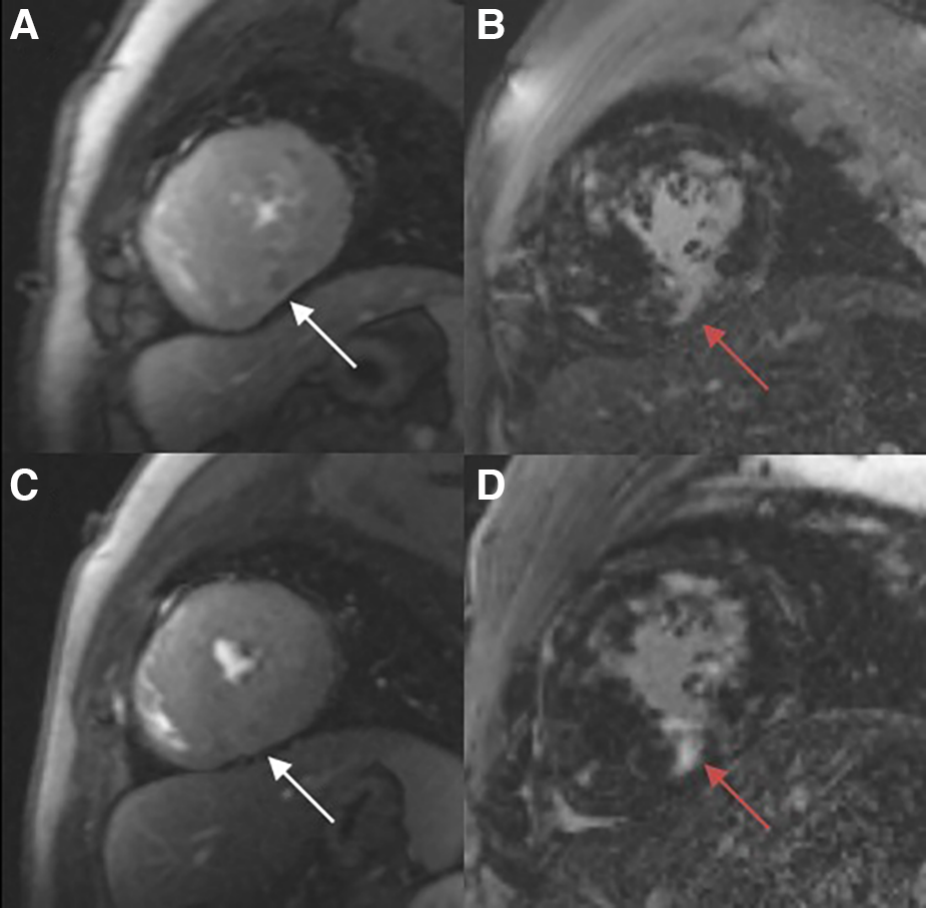
Myocardial perfusion examination (**A**, **C**) demonstrating a perfusion defect in the infer-septal midsegment on the images (white arrow). There are multiple patchy areas of late gadolinium enhancement (red arrow) in the myocardial segments with and without significant perfusion defects (**B**, **D**).

**Figure 5:**
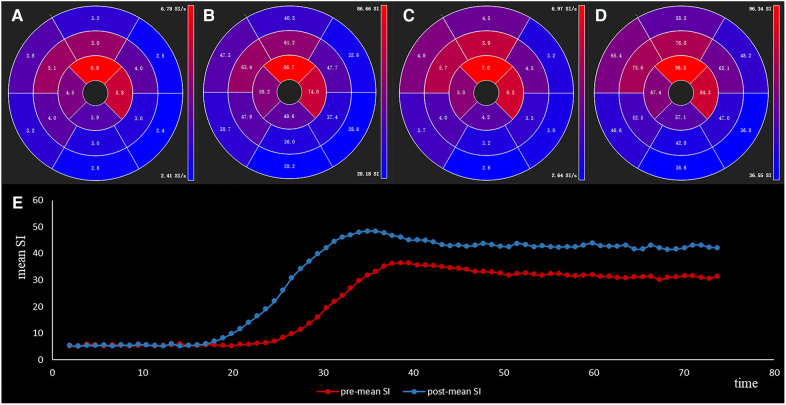
The slope_max_ (**A,C**) and sl_max_ (**B,D**) of sixteen segments of a 59-year-old male HOCM patient before and after TA-BSM. The lower image (**E**) shows the time-intensity curve preoperatively (in blue) and postoperatively (in orange).

The Slope_max_ and Sl_max_ were elevated, indicating that the blood filling rate and myocardial blood flow in the coronary microcirculation were significantly improved (Figures 3A,B). Similarly, the decrease in time_max_ means that the blood fully entered the cardiomyocytes more rapidly (Figure 3C). A representative case of myocardial perfusion before and after TA-BSM is shown in Figures 4, 5.

### Factors associated with the rate of change of slope_max_

**Figure 6:**
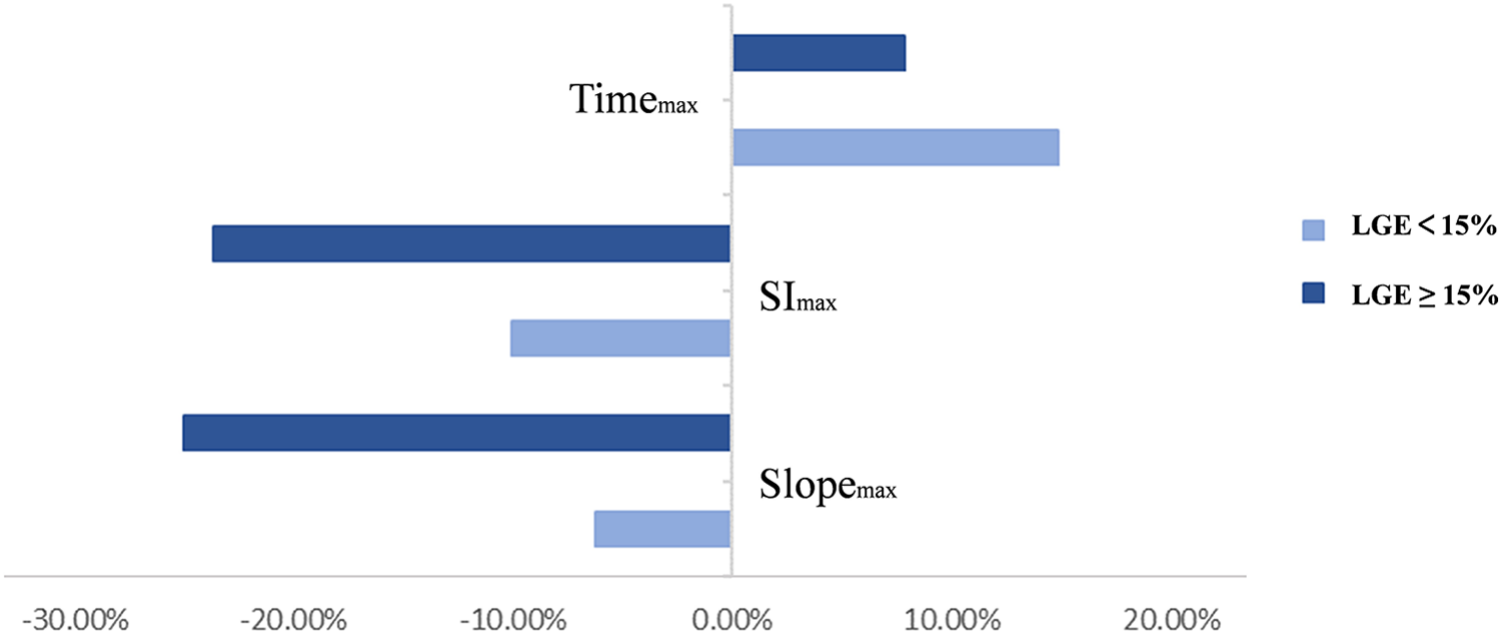
Two subgroups were divided depending on whether the left ventricular strengthening range was ≥15%. Rates of change in the perfusion parameters are shown in the graph above.

Patients were divided into two subgroups according to whether LGE was ≥15%. The rate of slope_max_ change in the LGE ≥ 15% group was significantly greater than in the LGE < 15% group (*p* = 0.002). However, there were no statistically significant differences in other perfusion parameters between the two subgroups (Figure 6). Given the collinearity of myocardial perfusion parameters, we only performed regression analysis for slope_max_ because it directly reflected the rate of blood flow filling of the myocardium. The results of the linear regression analysis are shown in Table 4. Age, weight of the resected myocardium, maximum wall thickness, LGE, and *Δ*LVOTG were significantly associated with the rate of slope_max_ change in the univariate and multivariate linear regression analyses (all *p *< 0.05).

**Table 4:** Univariate and multivariate analysis showing potential factors associated with the rate of slope_max_ change.

Variables	Univariate		Multivariate	
	r	*p*	Adjusted β	*p*
Age	0.518	<0.001	0.551	<0.001
Male	0.070	0.141		
Preoperative β-blockers	0.032	0.500		
Preoperative Diltiazem	0.078	0.154		
Rv5 + Sv1	−0.069	0.146		
Preoperative BNP	−0.170	<0.001	0.045	0.321
△6-minute walking distance (m)	0.071	0.213		
△Score of the Kansas City Cardiomyopathy Questionnaire	−0.022	0.649		
Weight of resected myocardium	−0.275	<0.001	0.191	0.001
Maximal wall thickness	−0.462	<0.001	−0.406	<0.001
LGE	−0.198	<0.001	0.260	<0.001
△Mitral regurgitation degree	−0.057	0.341		
△SAM	−0.111	0.058		
△LVOTG	0.108	0.023	−0.123	0.009

LGE, late gadolinium enhancement; SAM, systolic anterior motion; LVOTG, left ventricular outflow tract gradient.

### Inter- and intra-observer reproducibility

Inter- and intra-observer agreement for the perfusion parameters was high (*r* = 0.94 (0.89–0.97) and *r* = 0.95 (0.91–0.98), *p* < 0.05).

## Discussion

This study used cardiovascular magnetic resonance to quantitatively evaluate changes in myocardial microcirculation perfusion in HOCM patients after TA-BSM. The results showed that (1) LVOT obstruction was relieved after the operation, and the overall myocardial microcirculation perfusion of the left ventricle, including hypertrophic and non-hypertrophic myocardial segments, was significantly improved; (2) patients with severe fibrosis (LGE ≥ 15%) had more pronounced improvement in microcirculation after TA-BSM; and (3) in addition to age, weight of the resected myocardium, maximum wall thickness, LGE, and *Δ*LVOTG might be potential factors affecting the degree of improvement in the microcirculation after TA-BSM.

Myocardial ischemia is a significant pathophysiological feature in HCM patients without combined epicardial coronary stenosis (10–12) and is a multifactorial condition that includes microstructural disturbances and hemodynamic changes (2). The more common features include disorders of small artery architecture, small artery lesions, a mismatch between myocardial capillary density and increased LV myocardial mass, impaired coronary flow reserve, and imbalance between myocardial oxygen supply and demand (13–15). Inadequate oxygen supply in pathological left ventricular hypertrophy (LVH) leads to impaired energy metabolism in cardiomyocytes. Coronary blood flow is further impaired by inefficient contraction and diastolic dysfunction. Many studies also confirm that microcirculatory disorders play an essential role in the pathophysiological pathogenesis of myocardial ischemia in HCM (16, 17).

Micro-arterial remodeling is one of the mechanisms of microcirculatory dysfunction and plays an important role in the regulation of microcirculatory blood flow (15, 18). Vascular smooth muscle hypertrophy and endothelial cell proliferation result in small arterial lumen narrowing and diastolic restriction. This means less myocardial blood flow reserve, which may trigger local myocardial ischemia during accelerated heart rate, ultimately leading to fibrosis, heart failure, ventricular tachyarrhythmia and even SCD. Abnormal coronary anatomy, such as myocardial bridges, is common in HCM (19–21). This may contribute to the further deterioration of myocardial ischemia in HCM.

More than 90% of total resistance exists in vessels <300 *μ*m in diameter. In addition to intravascular resistance, extravascular compressive forces due to elevated left ventricular chamber pressure and wall stress caused by diastolic dysfunction and LVOT obstruction may also contribute to perfusion abnormalities (22, 23). This effect is mainly reflected in subendocardial perfusion injury in HCM. A study of eighteen patients with HCM showed that in addition to the extent of hypertrophy, the degree of LVOT obstruction and wall stress plays a critical role in microvascular dysfunction in HCM patients (24). This explains our findings that the reduction in LVOTG was significantly associated with improved microvascular function. Previous studies have shown that surgical myectomy and percutaneous alcohol ablation appears to increase coronary flow reserve in HOCM patients (25, 26). Compensatory hypertrophy due to increased afterload resolution after surgery might contribute to restoring capillary density, positively affecting microcirculatory perfusion (27).

Myocardial fibrosis and scar resulting in reduced myocardial blood flow and perfusion reserve and myocardial ischemia promotes fibrosis progression conversely. Dynamic coronary flow abnormalities of longer duration may exacerbate the progression of decompensation. According to a study of 35 HCM patients, perfusion reserve was reduced in proportion to the magnitude of hypertrophy, and the incidence of myocardial fibrosis decreased with increasing myocardial blood flow (28). A study of sixteen HCM patients showed a significant reduction in myocardial perfusion in the LGE (+) group; however, there was no statistically significant difference between the LGE (−) group and the normal control group (8).

Similar to the findings of previous studies, we found that maximum wall thickness and degree of fibrosis correlated with the rate of slope_max_ change, suggesting that a myocardial structural abnormality, particularly myocardial fibrosis, could play a significant role in microvascular dysfunction in HCM. Distinct from progressive reactive interstitial fibrosis, chronic or recurrent ischemic injury may promote collagen deposition, leading to additional fibrosis (29). This explains one of the mechanisms of adverse left ventricular remodeling. We also found a decrease in native T1 values postoperatively which may reflect a more subtle diffuse dilatation of the extracellular matrix caused by interstitial fibrosis, inflammation, edema and infiltrative processes (30). Additionally, the decreased T2 values may be related to the regression of chronic low-grade extracellular inflammation in postoperative reverse myocardial remodeling (31, 32), as evidenced by reversible myocardial edema and regression of collagen deposition (33, 34).

The study also showed that myocardial mass removed in TA-BSM was a potential factor affecting the degree of improvement in myocardial perfusion. Therefore, the quality and extent of ventricular septal myocardial resection requires individualized treatment for different patients.

The patient's LVEF decreased after TA-BSM, and the left ventricular hyperdynamic state was partially relieved. Our findings might provide new insights into the treatment of HCM. Currently, cardiovascular magnetic resonance research on myocardial perfusion in patients after surgery is limited, and the long-term effects of surgery on HCM patients require prolonged follow-up. Given the hemodynamic benefits, our study highlighted where and how much septal myocardium should be removed and will help improve the postoperative evaluation indicators.

There were several limitations in our study. First, the sample was small due to this new form of myectomy, but it included HOCM patients of different ages. Second, this minimally invasive procedure has not been performed in other healthcare settings, so we could only conduct a single-center study. Third, there may have been a selection bias because we excluded patients who did not receive regular follow-up with CMR. Fourth, exploration of microvascular dysfunction in relation to diastolic function and LGE patterns was not included in this study. Fifth, CMR assessment of tissue features was not validated by histological samples. Last, prognosis, which requires longer follow-up of postoperative patients, was not included in this study.

## Conclusions

Our study demonstrated improvement in coronary microcirculation in almost all myocardial segments in HOCM patients after TA-BSM, despite non-hypertrophic parts. After relieving the LVOT obstruction, the pressure load on the left ventricle decreased, and the blood perfusion rate and flow increased. Age, weight of the resected myocardium, maximum wall thickness, LGE, and *Δ*LVOTG might be potential factors suggesting improvement in the coronary microcirculation. Further studies are needed to demonstrate the long-term effects of TA-BSM on myocardial perfusion in HOCM.

## Data Availability

The raw data supporting the conclusions of this article will be made available by the authors, without undue reservation.
